# Altered Neural Reactivity in Adolescents With Nonsuicidal Self-Injury During Exposure to Self-Injury Related Cues: Electrophysiological Evidence From a Two-Choice Oddball Paradigm

**DOI:** 10.3389/fpsyt.2022.827480

**Published:** 2022-04-05

**Authors:** Dong-Dong Zhou, Lin Zhao, Ling-Li Ma, Jin-Hui Hu, Ran Chen, Zheng-Hao Jiang, Xiao-Qing He, Xin-Yu Peng, Xin-Yi Liu, Xiao Li, Wan-Jun Chen, Wo Wang, Li Kuang

**Affiliations:** ^1^Mental Health Center, University-Town Hospital of Chongqing Medical University, Chongqing, China; ^2^Department of Psychiatry, The First Affiliated Hospital of Chongqing Medical University, Chongqing, China

**Keywords:** nonsuicidal self-injury, event-related potentials, N2, P3, response inhibition, adolescent

## Abstract

**Background:**

Nonsuicidal self-injury (NSSI) may be a type of addiction, that is characterized by cue reactivity. We aimed to explore the behavioral performance and neural reactivity during exposure to self-injury cues in adolescents with NSSI and major depressive disorder (MDD).

**Methods:**

Eighteen MDD patients, 18 MDD patients with NSSI, and 19 healthy controls (HC) were recruited to perform a two-choice oddball paradigm. All subjects were 12–18 years old. Neutral cues and self-injury related cues separately served as deviant stimuli. Difference waves in N2 and P3 (N2d and P3d) were derived from deviant waves minus standard waves. Accuracy cost and reaction time (RT) cost were used as behavioral indexes, while the N2d and P3d were used as electrophysiological indexes; the N2d reflects early conflict detection, and the P3d reflects the process of response inhibition.

**Results:**

No significant main effects of group or cue or an effect of their interaction were observed on accuracy cost and P3d latency. For RT cost, N2d amplitude, and N2d latency, there was a significant main effect of cue. For P3d amplitude, there was a significant main effect of cue and a significant group × cue interaction. In the NSSI group, the P3d amplitude with self-injury cues was significantly larger than that with neutral cues. However, there was no such effect in the MDD and HC groups.

**Conclusions:**

Adolescents with NSSI showed altered neural reactivity during exposure to self-injury cue. Further studies with larger sample sizes are needed to confirm our results.

## Introduction

Nonsuicidal self-injury (NSSI) refers to deliberate self-injuring behaviors that lack suicidal intent. These behaviors often occur in adolescents. It was reported that the lifetime incidence of NSSI is ~17–60% (1). Notably, ~41.6% of adolescents with NSSI were diagnosed with major depressive disorder (MDD) (2). NSSI is also an independent risk factor for future suicide attempts and completed suicide (3). Thus, the negative consequences of NSSI cannot be ignored, although the behavior is generally not fatal.

Typically, people have the ability to inhibit impulsive or inappropriate behavior, which is called response inhibition or behavioral inhibitory control (BIC). The capacity to inhibit responses is important for better adaptation (4, 5). However, adolescents with NSSI repeatedly engage in self-injury behaviors, which suggests a lack of inhibitory control. A previous study demonstrated that executive function was abnormal in subjects with self-injury (6). The functional connectivity between the right orbitofrontal cortex and anterior cingulate gyrus is reduced in patients with self-injury (7). Based on a regional homogeneity (ReHo) analysis, the self-injury group had significantly lower ReHo values in brain areas such as the left fusiform gyrus, inferior frontal gyrus, left hippocampus, and bilateral parahippocampal gyrus than the healthy control group (6). These neuroimaging studies indicated abnormal brain activity in areas involved in response inhibition in subjects with self-injury.

Event-related potential (ERP) is a useful tool for investigating changes in brain activity. The psychological paradigms investigating response inhibition traditionally include go/nogo, stop-signal, and two-choice oddball paradigms (8). In the two-choice oddball paradigm, two types of stimuli are presented: standard stimuli (frequently presented) and deviant stimuli (infrequently presented). Subjects needed to differentially respond to the standard and deviant stimuli as quickly as possible. When deviant stimuli are presented, subjects need to inhibit their more frequent responses to standard stimuli and make the correct responses to deviant stimuli. In the go/nogo paradigm, only accuracy can serve as a behavioral index. There is no reaction time (RT) index for deviant stimuli because subjects do not respond to nogo stimuli (9). In contrast, the stop-signal paradigm can provide RT as a behavioral index but cannot provide accuracy (10). Compared with those two paradigms, the two-choice oddball paradigm provides both accuracy and RT for deviant stimuli, which provides a more comprehensive evaluation of the behavior index (8). Alternatively, the antisaccade task and emotional stop-signal task are also able to provide accuracy and response time (11–13).

The ERP components associated with response inhibition include N200 and P300, also referred to as N2 and P3. Generally, N2 is a negative wave that occurs ~200–300 ms after stimulus presentation. N2 represents early conflict detection and monitoring sourced from the anterior cingulate cortex (14). P3 is a positive wave that occurs ~300 ms after stimulus presentation. P3 represents the process of response inhibition control, sourced from the anterior cingulate cortex and lateral orbital frontal cortex (OFC) (9, 15).

Drug addiction is characterized by a recurring desire to continue taking a drug despite the resulting negative consequences. Behavioral addiction is similar to drug addiction but in different domains, such as food addiction and internet addiction (16). Similarly, NSSI may be regarded as a type of behavioral addiction (17). The emotional state experienced when patients with NSSI do not injure themselves is similar to the withdrawal state experienced by individuals who use drugs (18). Washburn et al. (19) pointed out that patients repeatedly engaging in NSSI have a strong desire for self-harming behavior. Moreover, self-harming behavior can result in the release of endogenous opioid peptides, which can ease the pain caused by the self-harm and result in pleasure and satisfaction (20). Similar to patients with NSSI, ERP, and neuroimaging studies have demonstrated that neural activity involved in response inhibition is altered in individuals with drug use disorders (21). Therefore, NSSI and addiction may have similar neurobiological underpinnings.

Drug addiction is characterized by cue reactivity (22), which has been demonstrated at both behavioral and neurobiological levels using different methodologies including ERP (23). The physiological and neurochemical changes during cue exposure may contribute to initiation and maintenance of drug use (23). An ERP study showed that the amplitude of the N2 difference wave (N2d) in heroin abstainers was significantly smaller than that in controls when exposed to drug-related cues. For heroin abstainers, the amplitude of the N2d wave with drug-related cue exposure was smaller than the amplitude with neutral cue presentation (24). These may suggest the early conflict detection is impaired during drug-related cue exposure. Additionally, the accuracy on nogo trials was decreased in smokers (25), and smoking-related cues may interfere with normal performance (26). This cue reactivity is not only exhibited in drug addiction but also in behavioral addiction, such as gambling, gaming, and buying disorders (27). Patients with pathological gambling had a higher accuracy on nogo trials during exposure to gambling related cues (28). Thus, behavioral performance and neural reactivity patterns may also be altered in adolescents with NSSI during exposure to self-injury related cues. In a previous study using a stop-signal task, self-injuring patients made more errors to negative emotional pictures, but made fewer errors to self-injury pictures (29). Another study suggested that impaired emotional response inhibition to self-injury related stimuli may be a risk factor for self-injury urges (30), thereby suggesting that cue reactivity may be a predictor for future self-injury behaviors.

Therefore, we hypothesized that adolescents with NSSI would have altered neural reactivity patterns and behavioral performance during exposure to self-injury cues. In this study, we aimed to explore the behavioral performance and neural reactivity during exposure to self-injury cues using the two-choice oddball paradigm in adolescents with NSSI.

## Methods

### Participants

Adolescents aged 12–18 years old, who had a current diagnosis of MDD, engaged in NSSI or not, and were previously untreated, were recruited from the outpatient department of University-Town Hospital of Chongqing Medical University and The First Affiliated Hospital of Chongqing Medical University. NSSI was determined by the criteria proposed in the Diagnostic and Statistical Manual of Mental Disorders, fifth edition (DSM-5) (31): in the most recent year, self-harm behaviors occurred at least five times. There was no restriction on gender. All subjects were right-handed, had normal or corrected normal vision, and had normal hearing. The exclusion criteria were as follows: (a) diagnosis of schizophrenia, bipolar disorder, mental retardation, obsessive-compulsive disorder, substance use disorder, or another primary diagnosis other than MDD; (b) previous suicide attempt; (c) previous head trauma or epilepsy; (d) pregnancy; and (e) chronic or severe physical conditions, such as thyroid disease, autoimmune disease, or heart and lung disease. Healthy controls (HC) were age-matched adolescents recruited via advertisement. They were also right-handed, had normal or corrected normal vision, and had normal hearing. They were not diagnosed with MDD and were not currently or previously injuring themselves. Other exclusion criteria were similar to those applied to the patients.

### Measures and Procedure

All subjects were interviewed using the MINI-International Neuropsychiatric Interview (M.I.N.I. KID 5.0) (32). Two well-trained psychiatrists evaluated them using the following scales: (a) Hamilton Depression Scale (HAMD) (33); (b) Barratt Impulsiveness Scale 11th version (BIS-11) (34); and (c) Ottawa self-injury inventory (OSI) (35).

**Figure 1:**
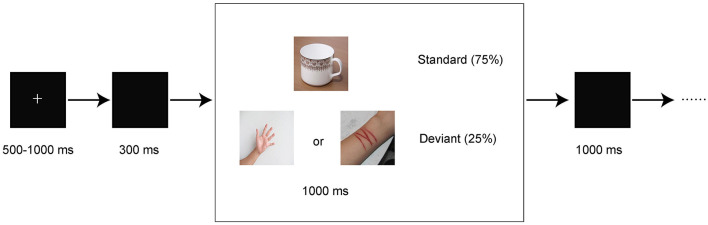
Schematic illustration of the experimental procedure and example stimuli. Each trial presented a single deviant or standard stimulus. Subjects pressed the “1” or “2” keys to respond to standard or deviant stimuli, respectively.

Subjects were seated in front of a computer screen at a distance of approximately 60 cm. E-Prime 3.0 was used to present stimuli. A two-choice oddball paradigm was used in our study. The paradigm is presented in Figure 1 and described as follows. Visual stimuli included frequent standard stimuli (75%) and infrequent deviant stimuli (25%). The paradigm included 2 conditions. One condition utilized neutral pictures as deviant stimuli, and the other condition utilized self-injury related pictures (cutting-related pictures) as deviant stimuli. In both conditions, a neutral cup was chosen as a standard stimulus. Each condition consisted of 200 trials, with a total of 400 trials included. In each trial, a fixation cross was initially presented for a randomly chosen duration between 500 and 1,000 ms, followed by a 300-ms blank screen. Then, a picture was presented for 1,000 ms or prematurely terminated by a response. Finally, a blank screen was presented for 1,000 ms. All pictures were chosen from free online resources and were retouched to the same size. The deviant stimuli were randomly chosen from 10 neutral pictures (neutral cues) and 10 self-injury related pictures (self-injury cues). The participants were required to press buttons based on the presented picture as quickly and accurately as possible. The subjects were asked to press the “1” key when a standard stimulus was presented and press the “2” key when a deviant stimulus was presented. The subjects needed to achieve at least 80% accuracy in practice before the formal trials were initiated.

### Acquisition and Preprocessing

A Neuroscan Quick cap with 64 scalp sites was used to record electroencephalography (EEG) data in the Curry 8 system. The impedance of each electrode was kept below 5 kΩ. The reference electrode between Cz and Cpz was chosen as an online reference. The sampling rate was 1,000 Hz, and the bandpass was 0.05–100 Hz. The accuracy and RT were recorded for each response. Offline analyses were conducted using the EEGLAB toolbox in MATLAB 2013b (36). We resampled all EEG data at 500 Hz. The offline bandpass filter was set at 0.1–30 Hz. All EEG data were re-referenced to the average of the M1 and M2 electrodes. EEG data were epoched to include 200 ms of the pre-stimulus and 1,000 ms of the post-stimulus periods. Epochs with an incorrect response were removed. Epochs with large artifacts were rejected, and bad channels were interpolated using the spherical method. Then, we ran independent component analysis (ICA) to remove artifact components, which mainly included blinking, horizontal eye movements, and some muscle artifacts (37). For each condition, epochs were overlapped and averaged separately. The 200-ms pre-stimulus baseline was subtracted by the post-stimulus wave to correct the baseline.

### Statistical Analysis

Three groups (HC, MDD, and MDD+NSSI) were included in our study. Regarding the behavioral indexes, accuracy cost was derived from the standard condition minus deviant condition, and RT cost was derived from the deviant condition minus standard condition. Two-way analysis of variance (ANOVA) was separately conducted for accuracy cost and RT cost. Cue served as the within-subject factor, and group served as the between-subject factor. Regarding the electrophysiological indexes, difference waves were derived from deviant waves minus standard waves. The peak latency and mean amplitude of N2d (200–300 ms) and P3d (350–550 ms) were extracted from the difference waves. N2 is typically largest in anterior areas, and P3 is typically largest in parietal or posterior areas (38–40). As in previous studies (41, 42), FCz was chosen as the electrode of interest for N2, and Cz was chosen as the electrode of interest for P3. Repeated measures ANOVA was performed for the mean amplitude and peak latency of N2d and P3d. The between-subject factor was the group (three levels: HC, MDD, and MDD+NSSI). The within-subject factor was the cue type (two levels: neutral cue and self-injury related cue). Greenhouse-Geisser correction was used when the sphericity assumption was violated. The significance level was set at *p* < 0.05. *Post hoc* comparisons were Bonferroni-adjusted. All the above analyses were performed using Statistical Package for Social Sciences (SPSS) version 25. We used the ERP reliability analysis (ERA) toolbox to obtain reliability estimates with a threshold of 0.70 (43, 44).

### *Post hoc* Analyses

Previous studies have suggested that impulsivity is associated with response inhibition (45). N2 represents early conflict detection and P3 represents the process of response inhibition. Thus, *post hoc* correlation analyses were performed between the BIS-11 total score and several variables (self-injury cue: P3d amplitude, P3d latency, N2d amplitude, N2d latency, RT cost, and accuracy cost; neutral cue: P3d amplitude, P3d latency, N2d amplitude, N2d latency, RT cost, and accuracy cost) for the three groups. Bonferroni corrections were used to correct for multiple comparisons.

## Results

### General Characteristics

We initially recruited 19 healthy controls, 19 MDD patients without NSSI, and 19 MDD patients with NSSI. One MDD patient and one NSSI patient were excluded from the analysis because of too many artifacts during EEG acquisition. The general characteristics of the three groups are presented in Table 1. Age and sex ratio were not significantly different across groups. However, BIS-11 total scores were significantly different among the groups [*F*_(2, 52)_ = 24.61, *p* < 0.001]. The BIS-11 total score in the HC group (33.72 ± 11.86) was significantly lower than that in the MDD (56.57 ± 13.59) and MDD+NSSI groups (63.05 ± 14.71) (Bonferroni-adjusted *p* < 0.001 for both). No significant BIS-11 total score difference was observed between the MDD and MDD+NSSI groups. No significant differences in the HAMD scores were found between the MDD and MDD+NSSI groups. The most frequent self-injury type was cutting (100%). A few adolescents also tried pinching, biting, knocking, or burning themselves. The reliability estimates are presented in Table 2. All reliability estimates were acceptable but NSSI group had a lower dependability for N2 latency in self-injury related deviant stimuli.

**Table 1:** Participant characteristics of the HC, MDD, and MDD+NSSI groups.

**Variable**	**HC (*n =* 19)**	**MDD (*n =* 18)**	**NSSI (*n =* 18)**	**Test statistic**
Age	16.11 (1.91)	16.00 (1.24)	15.17 (1.30)	F_(2, 52)_ = 2.08, *p* = 0.135
Female	57.9%	55.6%	83.3%	χ^2^ = 3.81; *p* = 0.149
percentage				
BIS-11 scores	33.72 (11.86)	56.57 (13.59)	63.05 (14.71)	*F*_(2, 52)_ = 24.61, *p* < 0.001
HAMD scores	–	21.61 (4.60)	23.56 (3.93)	t_(34)_ = −1.36, *p* = 0.182
**Injury type**
Cutting	–	–	18/18	–
Pinching	–	–	1/18	–
Biting	–	–	1/18	–
Burning	–	–	1/18	–
Knocking	–	–	1/18	–

*BIS-11, Barratt Impulsiveness Scale 11th version; HC, healthy controls; MDD, major depressive disorder; NSSI, nonsuicidal self-injury; HAMD, Hamilton Depression Scale*.

**Table 2:** Reliability estimates for electrophysiological and behavioral indices.

**Measurements**	**HC**	**MDD**	**NSSI**
**Neutral condition (standard stimuli)**			
N2 latency	0.90 (0.82, 0.96)	0.95 (0.90, 0.98)	0.88 (0.78, 0.95)
N2 amplitude	0.98 (0.97, 0.99)	0.95 (0.91, 0.98)	0.97 (0.95, 0.99)
P3 latency	0.95 (0.91, 0.98)	0.86 (0.76, 0.94)	0.93 (0.88, 0.97)
P3 amplitude	0.97 (0.95, 0.99)	0.96 (0.92, 0.98)	0.97 (0.94, 0.99)
Reaction time	0.98 (0.97, 0.99)	0.98 (0.96, 0.99)	0.98 (0.97, 0.99)
**Neutral condition (deviant stimuli)**			
N2 latency	0.75 (0.54, 0.89)	0.85 (0.73, 0.93)	0.83 (0.69, 0.92)
N2 amplitude	0.95 (0.91, 0.98)	0.87 (0.76, 0.94)	0.82 (0.67, 0.92)
P3 latency	0.73 (0.51, 0.88)	0.73 (0.51, 0.88)	0.86 (0.76, 0.94)
P3 amplitude	0.95 (0.92, 0.98)	0.95 (0.91, 0.98)	0.94 (0.89, 0.97)
Reaction time	0.98 (0.96, 0.99)	0.97 (0.96, 0.99)	0.98 (0.96, 0.99)
**Self-injury condition (standard stimuli)**			
N2 latency	0.88 (0.78, 0.94)	0.92 (0.86, 0.97)	0.91 (0.83, 0.96)
N2 amplitude	0.98 (0.96, 0.99)	0.96 (0.93, 0.98)	0.97 (0.94, 0.99)
P3 latency	0.93 (0.88, 0.97)	0.84 (0.71, 0.93)	0.88 (0.78, 0.95)
P3 amplitude	0.97 (0.95, 0.99)	0.96 (0.93, 0.98)	0.96 (0.94, 0.98)
Reaction time	0.98 (0.96, 0.99)	0.97 (0.95, 0.99)	0.97 (0.94, 0.99)
**Self-injury condition (deviant stimuli)**			
N2 latency	0.82 (0.67, 0.92)	0.83 (0.69, 0.93)	0.60 (0.26, 0.83)
N2 amplitude	0.95 (0.92, 0.98)	0.89 (0.81, 0.96)	0.89 (0.81, 0.95)
P3 latency	0.85 (0.74, 0.93)	0.84 (0.72, 0.93)	0.89 (0.80, 0.95)
P3 amplitude	0.94 (0.90, 0.97)	0.96 (0.93, 0.98)	0.93 (0.87, 0.97)
Reaction time	0.97 (0.95, 0.99)	0.97 (0.95, 0.99)	0.96 (0.93, 0.98)

*HC, healthy controls; MDD, major depressive disorder; NSSI, nonsuicidal self-injury*.

### Behavioral Indexes

A mixed ANOVA (group × cue) showed no significant main effect or interaction effect on accuracy cost. A mixed ANOVA (group × cue) for RT cost showed a significant main effect of cue [*F*_(1, 52)_ = 12.64, *p* = 0.001, $\eta_{\text{p}}^{2}$ = 0.20] but no significant interaction effect [*F*_(2, 52)_ = 0.56, *p* = 0.58, $\eta_{\text{p}}^{2}$ = 0.02] or main effect of group [*F*_(2, 52)_ = 1.38, *p* = 0.26, $\eta_{\text{p}}^{2}$ = 0.05].

### Electrophysiological Indexes

Regarding N2d amplitudes, a mixed ANOVA (group × cue) showed a significant main effect of cue [*F*_(1, 52)_ = 46.37, *p* < 0.001, $\eta_{\text{p}}^{2}$ = 0.47]. The main effect of group and the interaction effect were not significant. Regarding N2d latencies, a mixed ANOVA (group × cue) showed a significant main effect of cue [*F*_(1, 52)_ = 5.61, *p* = 0.022, $\eta_{\text{p}}^{2}$ = 0.10]. The main effect of group and the interaction effect were not significant.

**Figure 2:**
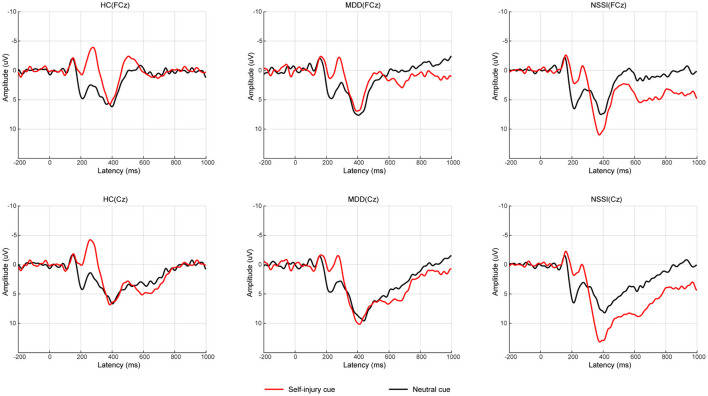
Average of difference ERPs for neutral cue condition and self-injury cue condition in HC group, MDD group, and NSSI group. ERP, event-related potential; HC, healthy control; MDD, major depressive disorder; NSSI, nonsuicidal self-injury.

**Figure 3:**
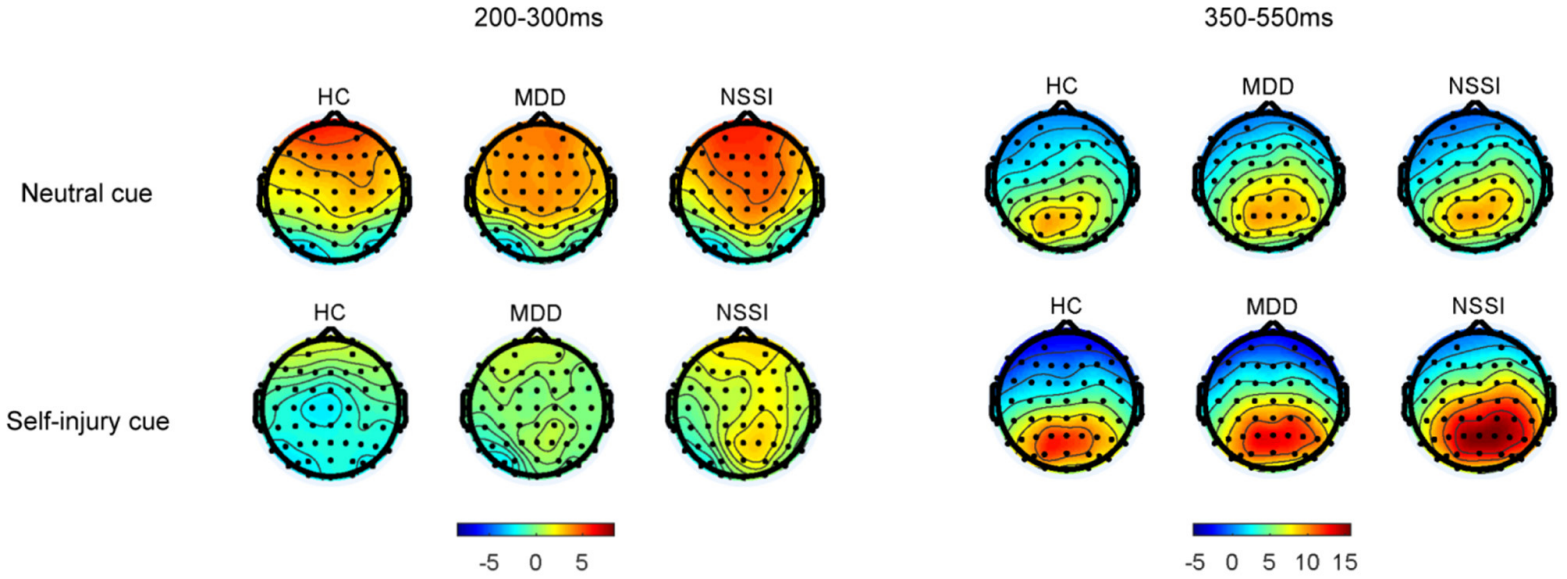
Grand-average topographic maps of the N2d (200–300 ms) and P3d (350–550 ms).

Regarding P3d amplitudes, a mixed ANOVA (group × cue) showed a significant main effect of cue [*F*_(1, 52)_ = 9.46, *p* = 0.003, $\eta_{\text{p}}^{2}$ = 0.15] and a significant group × cue interaction [*F*_(2, 52)_ = 5.15, *p* = 0.009, $\eta_{\text{p}}^{2}$ = 0.17]. The main effect of group was not significant. A simple effect analysis indicated that with self-injury cues, the P3d amplitude in the NSSI group was larger than that in the HC group (Bonferroni-adjusted *p* = 0.067). In the NSSI group, the P3d amplitude with self-injury cues was significantly larger than that with neutral cues (Bonferroni-adjusted *p* < 0.001). However, there was no such effect in the MDD and HC groups. Regarding P3d latencies, a mixed ANOVA (group × cue) showed no significant main effects or interaction effect. Grand mean ERPs were presented in Figure 2 and topographic maps of the N2d and P3d were presented in Figure 3.

### Correlation Analyses

For the three groups, correlation analyses were performed between the BIS-11 total score and several variables (self-injury cue: P3d amplitude, P3d latency, N2d amplitude, N2d latency, RT cost, and accuracy cost; neutral cue: P3d amplitude, P3d latency, N2d amplitude, N2d latency, RT cost, and accuracy cost). However, no *p*-value was significant after Bonferroni correction.

## Discussion

This study utilized a two-choice oddball paradigm to investigate the behavioral performance and neural reactivity during exposure to self-injury related cues in adolescents with NSSI. Three groups (NSSI+MDD, MDD, and HC) were compared. A simple effect analysis showed that the P3d amplitude with self-injury cues was significantly larger than that with neutral cues in the NSSI group (Bonferroni-adjusted *p* < 0.001). However, there was no such effect in the MDD and HC groups. These results suggest that the neural reactivity was altered during exposure to self-injury cues in adolescents with NSSI.

Regarding the behavioral indexes, no significant difference between groups was observed for accuracy cost. This result is consistent with previous studies and suggests little-to-no association between NSSI and behavioral performance (47, 48). For RT cost, the ANOVA revealed a significant main effect of cue, indicating that the RT cost with the self-injury cue was larger than that with the neutral cue regardless of group. Consistent with this finding, in a previous study, both the NSSI and control groups showed worse behavioral performance in response to self-injury related pictures than that in response to neutral pictures (29). Surprisingly, the NSSI group in that study showed significantly fewer errors during exposure to self-injury pictures, which was explained by an enhanced response inhibition to self-injury cues (29). Moreover, subjects with a different behavioral addiction, pathological gambling, also showed an enhanced response inhibition during exposure to gambling related cues (28). However, these effects were not observed in our study, which possibly due to differences in recruited participants or paradigms. Only adolescents were recruited in our study, while only adults with NSSI were recruited in a previous study (29). Further studies are needed to confirm this effect.

Regarding the electrophysiological indexes, only the main effect of cue was significant for N2d amplitudes and N2d latency. The N2d represents conflict detection and monitoring (8). The significant main effect of cue may suggest general detection of a larger conflict during exposure to self-injury cues regardless of group. The P3d represents the process of response inhibition. The P3d amplitude in the NSSI group was larger than that in the HC group with self-injury related cues, which suggested that adolescents with NSSI needed more neural resources to successfully complete the inhibition. This result was consistent with some studies. Alcohol-related cues induced larger P3 amplitudes in heavy drinkers than in light drinkers (49). Consistently, a heroin addiction group showed a larger P3d amplitude than controls (50). The internet addiction disorder (IAD) group also showed a larger P3 amplitude than controls (51). However, some studies have reported that a lower P3 amplitude reflects a deficient capability of inhibition (52). Smaller P3 amplitudes were observed in heavy drinkers than in light drinkers (46). Smokers had a reduced P3 amplitude compared with non-smokers (42). Additionally, P3 amplitude was negatively associated with nogo errors, suggesting that subjects with reduced P3 amplitude had more errors (42). These studies seem to be inconsistent with our results. Through compensatory mechanisms, binge drinkers may increase nogo P3 amplitudes and produce a greater activation in prefrontal areas, to achieve a performance level comparable to controls (53). In our study, the NSSI group may be compensating to increase their P3d amplitude to achieve a similar accuracy as the HC group. These results also suggested that the inhibitory capacity was impaired in adolescents with NSSI.

In the NSSI group, the P3d amplitude with self-injury related cues was significantly larger than that with neutral cues. No such effect was observed in the MDD or HC groups. Thus, similar to individuals with addiction disorders, adolescents with NSSI may have an altered neural reactivity when exposed to self-injury cues. Addicted subjects showed altered brain activation to drug-related cues, involving the salience, executive, and reward networks (54). Compared with the controls, subjects with NSSI in a previous study showed enhanced activity in the inferior frontal cortex (IFC) and OFC when exposed to self-injury cues (55). This neuroimaging result is consistent with our results; both indicate that adolescents with NSSI may process self-injury cues differently. Drug-related cues are more salient to addicted subjects than neutral cues (56, 57). Likewise, self-injury cues may be more salient to subjects with NSSI, resulting in more neural resources being needed to inhibit inappropriate responses when exposed to self-injury cues.

We should note that both P3 amplitude reductions and increases have been inconsistently reported in previous studies (42, 46, 49–51, 57, 58). It appears to be very confusing that both P3 reductions and increases can indicate deficiency in response inhibition. In our opinion, behavioral and electrophysiological indexes should be used simultaneously to evaluate response inhibition. Here, we propose two concepts: subthreshold and suprathreshold deficiency in response inhibition. Suprathreshold deficiency represents behavioral performance that was significantly worse than performance in controls (i.e., accuracy was lower and RT was longer), while subthreshold deficiency represents behavioral performance that was similar to performance in controls. Usually, reduced P3 amplitudes can be observed in instances of suprathreshold deficiency (42, 58). Increased P3 amplitudes may accompany subthreshold deficiency, suggesting that subjects use more neural resources to achieve a similar behavioral performance (51). Suprathreshold deficiency accompanied by a larger P3 amplitude may indicate a worse inhibitory capacity, such as cases where subjects have worse performance although more neural resources are used (49, 50).

This study had some limitations. First, although we did not find an overall sex difference among the groups, there were more female participants in the MDD+NSSI group (83.3%). This may be because females are more likely to engage in NSSI (1). The NSSI group was younger than the other groups, although this difference was not significant. Thus, studies with larger sample sizes and groups that are more balanced in terms of sex and age are required in the future. Our results should be treated with caution. Second, all subjects with NSSI cut themselves, and only cutting-related pictures were used as self-injury cues. Our results thus may not be generalizable to subjects with other forms of self-injury. Third, this was a cross-sectional study; thus, it remains unclear whether altered neural reactivity during exposure to self-injury cues predicts future self-injury or suicidal behavior. The deficits in inhibitory control with self-injury cues may be a risk factor for self-injury urges (30), suggesting that cue reactivity may be a predictor for NSSI in the future. Further longitudinal studies are needed. Fourth, all adolescents with NSSI in our study were diagnosed with MDD; thus, our results may not apply to adolescents without MDD or individuals with other diagnoses.

## Conclusions

Our study provided electrophysiological evidence that neural reactivity in adolescents with NSSI was altered during exposure to self-injury cues. Specifically, the P3d amplitude was larger during exposure to self-injury cues. Studies with larger sample sizes are needed to confirm our results.

## Data Availability Statement

The raw data supporting the conclusions of this article will be made available by the authors, without undue reservation.

## Ethics Statement

This study was approved by the Ethics Committee of the University-Town Hospital of Chongqing Medical University. Written informed consent to participate in this study was provided by the participants' legal guardian/next of kin.

## Author Contributions

D-DZ, RC, and LK were involved in the study concept and design. LZ, L-LM, J-HH, Z-HJ, X-QH, X-YP, X-YL, XL, W-JC, and WW were involved in data preparation and interpretation. D-DZ, LZ, L-LM, X-QH, and J-HH were involved in the statistical analysis. D-DZ, RC, and Z-HJ wrote the manuscript. D-DZ and LK were involved in study supervision and edited the manuscript. All authors had full access to all data in the study and take responsibility for the integrity of the data and the accuracy of the data analysis.

## Funding

This work was supported by a grant from the Science and Technology Bureau of Chongqing (cstc2020jcyj-msxmX0222) and a grant from the National Natural Science Foundation of China (81971286).

## Conflict of Interest

The authors declare that the research was conducted in the absence of any commercial or financial relationships that could be construed as a potential conflict of interest.

## Publisher's Note

All claims expressed in this article are solely those of the authors and do not necessarily represent those of their affiliated organizations, or those of the publisher, the editors and the reviewers. Any product that may be evaluated in this article, or claim that may be made by its manufacturer, is not guaranteed or endorsed by the publisher.
